# Developments of the Physical and Electrical Properties of NiCr and NiCrSi Single-Layer and Bi-Layer Nano-Scale Thin-Film Resistors

**DOI:** 10.3390/nano6030039

**Published:** 2016-02-25

**Authors:** Huan-Yi Cheng, Ying-Chung Chen, Chi-Lun Li, Pei-Jou Li, Mau-Phon Houng, Cheng-Fu Yang

**Affiliations:** 1 Department of Electrical Engineering, National Sun Yat-sen University, Kaohsiung 804, Taiwan; eveflora818@gmail.com (H.-Y.C.); ycc@mail.ee.nsysu.edu.tw (Y.-C.C.); insist256@hotmail.com (C.-L.L.); sunnyrain516@hotmail.com (P.-J.L.); 2 Institute of Microelectronics, National Cheng-Kung University, No.1, University Road, Tainan City 701, Taiwan; mphoung@eembox.ncku.edu.tw; 3 Department of Chemical and Materials Engineering, National University of Kaohsiung, Kaohsiung 81141, Taiwan

**Keywords:** thin-film resistor, sputtering method, sheet resistance, value of temperature coefficient of resistance (TCR value), Bi-layer

## Abstract

In this study, commercial-grade NiCr (80 wt % Ni, 20 wt % Cr) and NiCrSi (55 wt % Ni, 40 wt % Cr, 5 wt % Si) were used as targets and the sputtering method was used to deposit NiCr and NiCrSi thin films on Al_2_O_3_ and Si substrates at room temperature under different deposition time. X-ray diffraction patterns showed that the NiCr and NiCrSi thin films were amorphous phase, and the field-effect scanning electronic microscope observations showed that only nano-crystalline grains were revealed on the surfaces of the NiCr and NiCrSi thin films. The log (resistivity) values of the NiCr and NiCrSi thin-film resistors decreased approximately linearly as their thicknesses increased. We found that the value of temperature coefficient of resistance (TCR value) of the NiCr thin-film resistors was positive and that of the NiCrSi thin-film resistors was negative. To investigate these thin-film resistors with a low TCR value, we designed a novel bi-layer structure to fabricate the thin-film resistors via two different stacking methods. The bi-layer structures were created by depositing NiCr for 10 min as the upper (or lower) layer and depositing NiCrSi for 10, 30, or 60 min as the lower (or upper) layer. We aim to show that the stacking method had no apparent effect on the resistivity of the NiCr-NiCrSi bi-layer thin-film resistors but had large effect on the TCR value.

## 1. Introduction

A wide variety of materials have been investigated as thin-film resistors in integrated circuit (IC) applications. The need for appropriate properties—such as high sheet resistance, low-temperature coefficient of resistance, and stability under ambient conditions—has motivated investigations into electronic conduction mechanisms in a number of ceramal [1,2] and alloy resistor systems [3,4]. In IC fabrication technologies, resistors can be implemented by using diffusion methods fabricated in the base and emitter regions of bipolar transistors, or in the source/drain regions of a CMOS, or by depositing thin films on the surfaces of wafers. Numerous studies have been published on the active metal brazing of engineering ceramics to increase service temperatures [5]. Thompson *et al.* investigated the magnetic properties of a series of NiCr alloys, which possess lower Curie temperatures and lower saturation magnetization. Those alloys have the potential to be developed as suitable alloys for reducing ferromagnetism, and can be biaxially textured [6,7].

NiCr alloys have also for many years been the most common materials as using in metal-based thin-film resistors, so considerable attentions have been focused on understanding the resistivity and temperature coefficient of resistance (TCR) of vacuum-deposited thin films [8,9]. For that, NiCr (80 wt % Ni, 20 wt % Cr) thin films were prepared by radio frequency (RF) magnetron sputter method because the deposition technology was used widely in thin films’ preparation, and the NiCr thin films were deposited on silicon (Si) and Al_2_O_3_ substrates under different deposition time. CrSi-based thin films are a particularly interesting material to be deposited as the thin-film resistors, as they offer certain advantages [10]. For example, they have high sheet resistance, low TCR values, high thermal stability, good long-term reliability, and chemical stability [11]. Dong and colleagues investigated the properties of CrSi (Cr:Si = 1:3) thin-film resistors and found that the resistors doped with 3–6 at % Ni had resistivity 1.31–1.49 times higher than those of CrSi thin films without Ni [12]. Lee and Shin also investigated NiCr-based alloy thin-film resistors with the precision characteristics, using a material comprised of Ni (75 wt %), Cr (20 wt %) Al (3 wt %), Mn (4 wt %), and Si (1 wt %) as target, a DC magnetron as sputtering method, and changing the various deposition parameters, such as power, pressure, substrate temperature, and post-deposition annealing temperature [13].

In the present study, we employed an RF magnetron sputtering method to deposit NiCr (80 wt % Ni, 20 wt % Cr) and NiCrSi (55 wt % Ni, 40 wt % Cr, 5 wt % Si) thin films on Si and Al_2_O_3_ substrates using different deposition time. We found that even in the amorphous phase, the thicknesses of the NiCr and NiCrSi thin films increased with increasing deposition time. We thoroughly investigated the effects of deposition time on the electrical properties of the NiCr and NiCrSi thin films, and the measured results suggested that deposition time affected their properties, including resistance, resistivity, and TCR. We also found that NiCr thin-film resistors had positive TCR values whereas NiCrSi thin-film resistors had negative TCR values. We therefore examined thin-film resistors with two different bi-layer NiCr and NiCrSi thin-film structures. To create the bi-layers, we fixed the deposition time of the NiCr thin films at 10 min and the deposition time of the NiCrSi thin films at 10, 30, or 60 min. We would show that they could be explored to fabricate the thin-film resistors having TCR values close to 0 ppm/°C. We also showed that the deposition conditions (*i.e.*, whether the NiCr thin films had been deposited as upper or lower layers) and the deposition time (and, hence, the thickness) of the NiCrSi thin films in the bi-layer structures had a large effect on the electrical properties of our NiCr-NiCrSi bi-layer thin-film resistors.

## 2. Experimental Section

**Figure 1:**
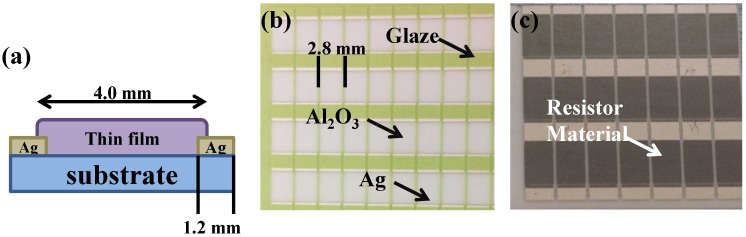
(**a**) Schematic side view of the thin-film resistors’ structure, (**b**) top view before the protective glass films (green color) were removed, and (**c**) top view after the protective glass films were removed and the resistor materials (black) were deposited.

In this study, we used commercial-grade NiCr (80 wt % Ni, 20 wt % Cr) and NiCrSi (55 wt % Ni, 40 wt % Cr, 5 wt % Si) as the targets. We then employed the RF magnetron sputtering method to deposit NiCr and NiCrSi thin films on Al_2_O_3_ substrates for electrical and thickness measurements and on Si substrates for cross-sectional observation and thickness measurement. The side view of the deposited single-layer NiCr-based and NiCrSi-based thin films is shown in Figure 1a, which is used to measure the physical and electrical properties. A green glass paste on the ceramic, shown in Figure 1b, was used to protect the Al_2_O_3_ substrate and it was removed after the deposition processes, following ultrasonic cleaning in ethyl alcohol. The length between the two electrodes (also called the length of the thin-film resistors) was 4.0 mm, the width of the thin-film resistors and electrodes was 2.8 mm, and the length of the electrodes was 1.2 mm (see Figure 1a,b). The deposition parameters were: deposition power 150 W, deposition pressure 5 mTorr in a pure Ar atmosphere, and deposition temperature 25 °C. The deposition time was changed from 10 min to 60 min for the NiCr-based thin films and from 10 min to 150 min for the NiCrSi-based thin films. A surface macro image of the fabricated NiCrSi thin-film resistors is shown in Figure 1c. The crystalline structures of the deposited NiCr-based and NiCrSi-based thin films were determined by X-ray diffraction (XRD) (Cu-Kα, Bruker D8, Billerica, MA, USA). The thicknesses of the as-deposited single-layer NiCr and NiCrSi thin films were obtained using α-step equipment and confirmed using field emission scanning electron microscopy (FESEM, Hitachi, Osaka, Japan). FESEM was also employed to determine the surface morphologies of the deposited NiCr-based and NiCrSi-based thin films and to obtain cross-sectional observations and confirm the thicknesses of the bi-layer thin-film structures.

The resistances of the NiCr and NiCrSi thin-film resistors were measured using the four-point probe method, and the resistivity was calculated with the measured resistances and thicknesses of the NiCr and NiCrSi thin films, according to Equation (1):

*R* = ρ × *A*/*l* (1)

where *R* is the resistance, ρ is the resistivity, *A* is the area of the resistor, and *l* is the length of the resistor.

**Figure 2:**
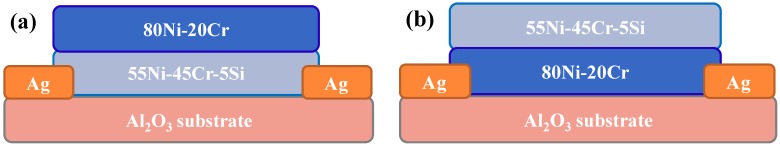
Schematic structures of two different bi-layer NiCr and NiCrSi thin-film resistors. (**a**) NiCr thin films were deposited for 10 min as the upper layer, and NiCrSi thin films were deposited for 10, 30, or 60 min as the lower layer; (**b**) NiCr thin films were deposited for 10 min as the lower layer, and NiCrSi thin films were deposited for 10, 30, or 60 min as the upper layer.

In this study, the measured temperatures were 25, 50, 75, 100, and 125 °C. The resistivity measured at those temperatures was used to find the TCR values of the NiCr-based and NiCrSi-based thin-film resistors and the two bi-layer thin-film resistors. As already mentioned, we found that the NiCr-based thin-film resistors had positive TCR values whereas the NiCrSi-based thin-film resistors had negative TCR values, so we investigated novel NiCr-NiCrSi bi-layer thin-film resistors, the structures of which are shown in Figure 2. We hoped to develop a thin-film resistor with a TCR value close to 0 ppm/°C, so we created the bi-layer thin-film resistors using two different stacking methods: (i) NiCr thin films were deposited for 10 min as the upper layer, and NiCrSi thin films were deposited for 10, 30, or 60 min as the lower layer (Figure 2a); or (ii) NiCr thin films were deposited for 10 min as the lower layer and NiCrSi thin films were deposited for 10, 30, or 60 min as the upper layer (Figure 2b).

To measure each layer’s and bi-layer’s thicknesses, at least eight samples were deposited at the same time. After deposition, at least three samples were used to measure the thickness of the lower layer, which was also obtained using α-step equipment and confirmed with FESEM. The other five samples were used to deposit the upper layer, and the total thickness of the bi-layer thin films was also measured by the same process. The thickness of the upper layer was calculated using the total thickness minus the thickness of the lower layer and was confirmed by FESEM. The resistance of the bi-layer thin-film resistors was also measured using the four-point probe method, and the resistivity was calculated from the resistance and thickness of the bi-layer structures’ thin films. Finally, we measured the TCR of the bi-layer structures’ thin-film resistors.

## 3. Results and Discussion

**Figure 3:**
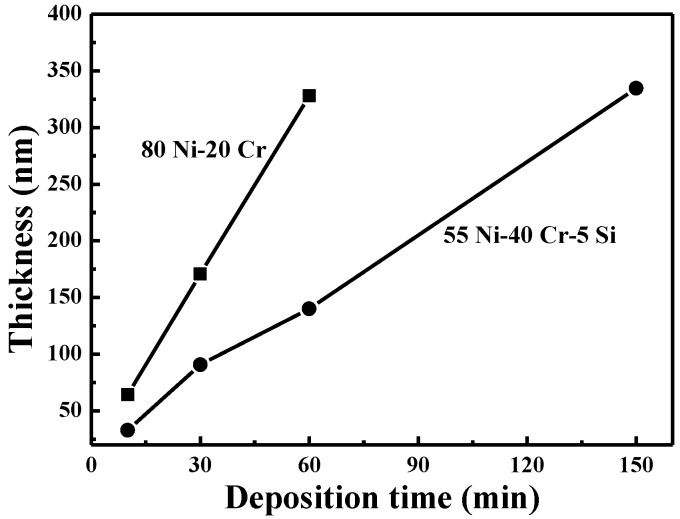
Variations in the thicknesses of as-deposited NiCr and NiCrSi thin-film resistors as a function of deposition time.

The effect of deposition time on the thicknesses of the NiCr-based and NiCrSi-based thin films was investigated, and the results are shown in Figure 3. The thicknesses of the NiCr thin films deposited over 10, 30, and 60 min were about 64.3, 170.7, and 327.9 nm, and the thicknesses of the NiCrSi thin films deposited over 10, 30, 60, and 150 min were about 30.8, 90.7, 140.1, and 334.7 nm, respectively. The results in Figure 3 suggest that the deposition rate of the NiCr thin films was higher than that of the NiCrSi thin film. In both instances, however, as the deposition time increased, there was a linear increase in the thicknesses of the NiCr-based and NiCrSi-based thin films.

**Figure 4:**
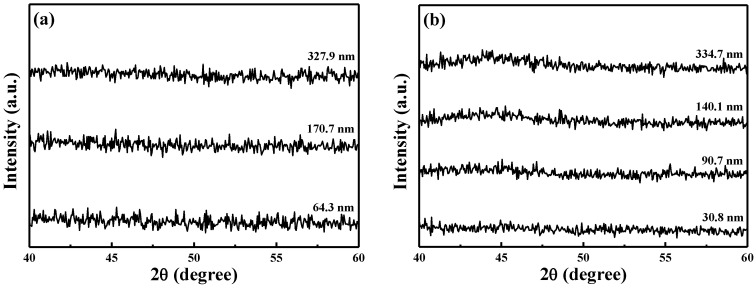
X-ray diffraction (XRD) patterns of 25 °C-deposited (**a**) NiCr and (**b**) NiCrSi thin-film resistors as a function of the thin films’ thickness (or deposition time).

**Figure 5:**
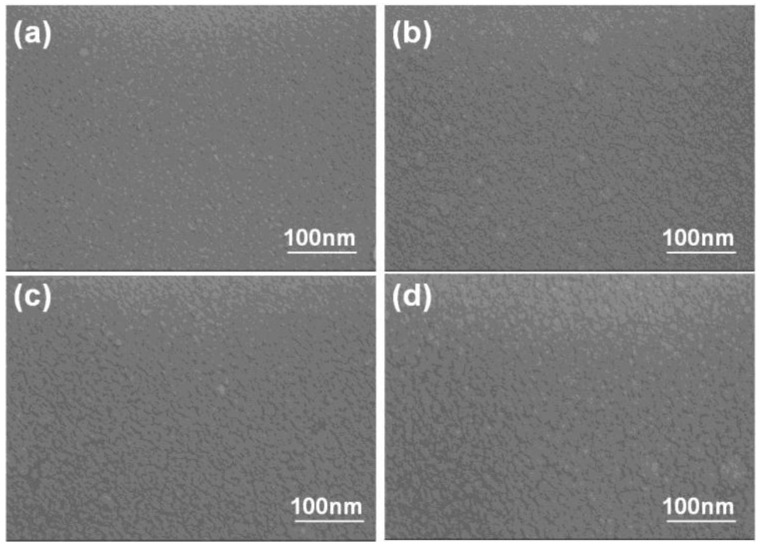
Surface morphologies of 25 °C-deposited thin films as a function of deposition time. NiCr thin films deposited at (**a**) 10 min and (**b**) 60 min. NiCrSi thin films deposited at (**c**) 10 min and (**d**) 150 min.

XRD was used to investigate the crystalline properties of the NiCr and NiCrSi thin films at room temperature. All of the XRD patterns (Figure 4) of the NiCr and NiCrSi thin films revealed the amorphous structure, and no crystalline phases were apparently observed, and only the Ag and Al_2_O_3_ phases were observed (not shown here). These results suggested that the thickness (or deposition time) had no effect on the crystallization of the as-deposited NiCr and NiCrSi thin films. Because the NiCr and NiCrSi thin films were deposited using a sputtering method in a pure Ar atmosphere, we believe that oxidation did not occur during the deposition process. We used FESEM to observe the surface morphologies of the NiCr thin-film resistors (see Figure 5a for deposition time of 10 min and Figure 5b for that of 60 min) and of the NiCrSi thin-film resistors (see Figure 5c for deposition time of 10 min and Figure 5d for that of 60 min). Only nano-crystalline grains were observed, and the surface morphologies were almost unchanged, regardless of the deposition time.

**Figure 6:**
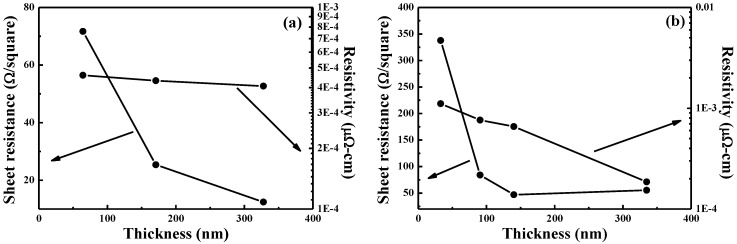
Variations in the resistance and resistivity of 25 °C-measured as-deposited (**a**) NiCr and (**b**) NiCrSi thin-film resistors as a function of the thin films’ thickness.

Figure 6 shows the effects of thickness (deposition time) on resistance and resistivity for NiCr and NiCrSi thin-film resistors measured at 25 °C. The resistances of the NiCr and NiCrSi thin-film resistors were recorded by the four-point probe method, and resistivity was derived from resistance using a measurement of the thin films’ thicknesses, shown in Figure 3. As the temperature increased from 25 to 125 °C, the resistance of the NiCr thin-film resistors slightly decreased and that of the NiCrSi thin-film resistors slightly increased. The NiCr and NiCrSi thin-film resistance values at 25 and 125 °C were similar. Figure 6a shows that the resistance of the NiCr thin-film resistors monotonously decreased as the thin films’ thickness increased. Figure 6b also shows that the thinner NiCrSi thin-film resistors showed higher resistance, and the resistance reached a saturation value as the thin films’ thickness became equal to or greater than 140.1 nm (*i.e.*, when the deposition time was equal to or greater than 60 min). If we suppose that the thicknesses of the NiCr and NiCrSi thin-film resistors were independent of the measured temperature, then the resistivity of the NiCr and NiCrSi thin-film resistors at 25 and 125 °C were similar and the variations in resistivity were not apparently observed.

In the past, only a few papers have discussed the effects of thin films’ thickness on their resistance and resistivity, as it has been difficult to discern a correlation between these values. In the free-electron model of metallic thin-film resistors with hard-wall boundary conditions, the discretization of energy levels makes it impossible to treat both the Fermi energy and the electron density as independent of thickness [14]. In this study, as the thin films’ thicknesses increased, the log(resistivity) of the NiCr (Figure 6a) and NiCrSi (Figure 6b) thin-film resistors linearly decreased. Katumba and Olumekor found that the log(ρ) of Cu-MgF_2_ cermet thin-film resistors’ thickness exhibited an approximately linear decrease from about 110 to 300 nm. Ultimately, they found the relationship between resistivity and thickness for thin-film Cu-MgF_2_ cermets to be as follows [2]:

ρ_f_ = ρ_o_ × exp(10 × *S/t*) (2)

where ρ_f_ is the resistivity of thin films, ρ_o_ is the limiting resistivity of very thick cermets, *t* is the film thickness, and *S* is a measure of the separation between the metallic islands embedded in the insulator matrix of the cermets. The present study not only sought the qualitative effects of the thicknesses of the NiCr and NiCrSi thin-film resistors but also attempted to quantify the relationships between resistivity and thickness. We found that the log(resistivity) values of the NiCr and NiCrSi thin-film resistors in Figure 6 decreased in an approximately linear mode as their thicknesses increased—similar to Cu-MgF_2_ cermet thin-film resistors.

Resistances for materials at any temperature other than standard temperature (usually taken to be 20 °C) on the specific resistance can be determined using the following formula:

*R* = *R*_ref_ [1 + α (*T* – *T*_ref_)] (3)

where *R* is the material resistance at temperature *T*; *R*_ref_ is the material resistance at temperature *T*_ref_, usually 20 °C or 0 °C; α is the TCR value for the material, symbolizing the resistance change factor per degree of temperature change; *T* is the material temperature in degrees Celsius; and *T*_ref_ is the reference temperature that α is specified at for the material.

**Figure 7:**
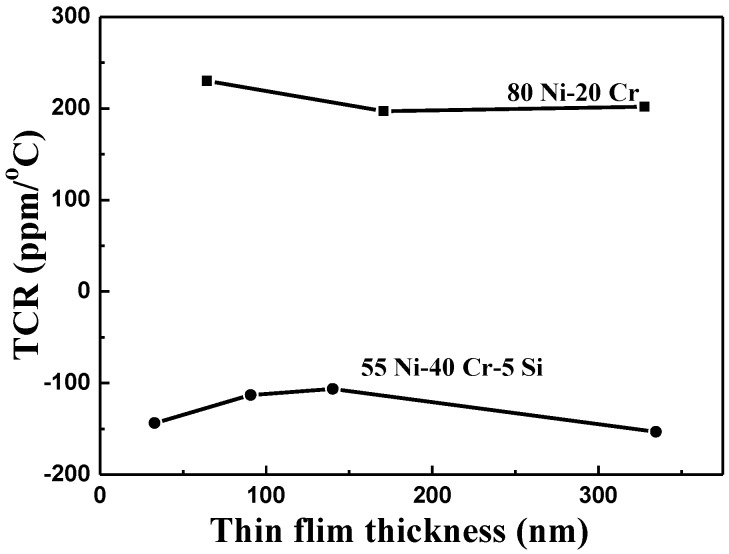
Variations in the temperature coefficient of resistance of as-deposited NiCr and NiCrSi thin-film resistors as a function of the thin-films’ thickness.

The TCR values of as-deposited NiCr and NiCrSi thin-film resistors are shown in Figure 7 as a function of the thin films’ thicknesses, using the measured results shown in Figure 6. For most pure metals, these TCR values are positive. Dhere *et al.* found that the NiCr thin-film resistors with low positive TCRs (*i.e.*, fewer than 100 ppm/°C) had been obtained at all thicknesses studied when the total atomic content of chromium, oxygen, and carbon reached 50%–55% [15]. The TCR value of Ni metal is 0.00017 and of Cr metal is 13 × 10^−8^, meaning that resistance increases with increasing measured temperature. Nevertheless, the TCR value of as-deposited NiCr thin-film resistors was positive in the range of 197.2 to 230.1 ppm/°C, which are larger than those of Ni and Cr metals. Single crystalline Si has a TCR value of about −0.04 (depending strongly on the presence of impurities in the material), so Si could be added to change the TCR value of NiCr-based thin-film resistors. As Figure 7 shows, the TCR values of as-deposited NiCrSi thin-film resistors were negative, in the range of −106.4 to −153.3 ppm/°C, meaning that the resistance decreased as the measured temperature increased. The TCR value of the NiCrSi thin-film resistors, shown in Figure 7, had no significant change as the thin-film’s thickness increased from 30.8 nm to 334.7 nm. Ni and Cr are metals, and Si is a semiconductor, and the XRD patterns in Figure 4 show that the NiCrSi thin films were in the amorphous phase. Those results suggest that as the NiCrSi thin films are deposited, the Ni and Cr will form alloys and then NiCr alloys will form a NiCrSi compound with Si. The electrical properties of a compound are the sum total of each component in it. Hence, the electrical properties of Si would affect the TCR value of the NiCrSi thin-film resistors. Ni and Cr, as well as NiCr thin-film resistors, have the positive TCR values. We believe that the negative TCR value of the NiCrSi thin-film resistors was caused by the addition of Si into the NiCr alloy.

**Figure 8:**
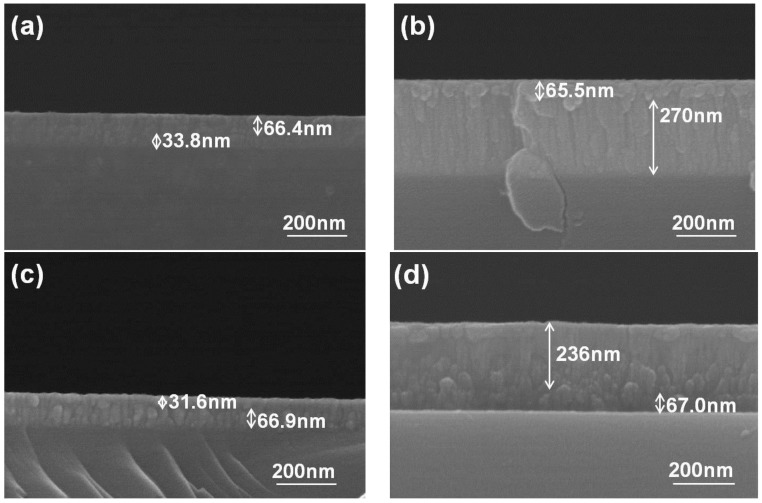
Cross-sectional images of the bi-layer thin-film resistors created using different deposition time of the NiCrSi thin films. In (**a**,**b**), a NiCr thin film deposited for 10 min was used as the upper layer, and a NiCrSi thin film deposited for (**a**) 10 min or (**b**) 60 min was used as the lower layer. In (**c**,**d**), a NiCr thin film deposited for 10 min was used as the lower layer, and a NiCrSi deposited thin film deposited for (**c**) 10 min or (**d**) 60 min was the upper layer.

As Figure 6 shows, the resistance of NiCr thin-film resistors linearly decreased as their thickness increased with deposition time, and the resistance of the NiCrSi thin-film resistors was almost unchanged as the thickness became equal to or greater than 140.1 nm (10 min deposition time). To simplify the fabrication process, the thickness (deposition time) of the NiCr thin films was fixed at 64.3 nm (10 min), and the thickness (deposition time) of the NiCrSi thin films was set at 30.8 nm (10 min), 90.7 nm (30 min), and 140.1 nm (60 min), respectively. Cross-section images of the as-deposited bi-layer thin-film resistors with their various structures and with the different NiCrSi thin films’ thicknesses are presented in Figure 8, where the bi-layer structure is easily observed. Figure 8 shows that the thickness of the NiCr thin films was in the range of 65.5–67.0 nm, which is similar to the value obtained from the single-layer thin films shown in Figure 3. Whether it was being used as the upper layer or the lower layer, it almost had the same value.

**Figure 9:**
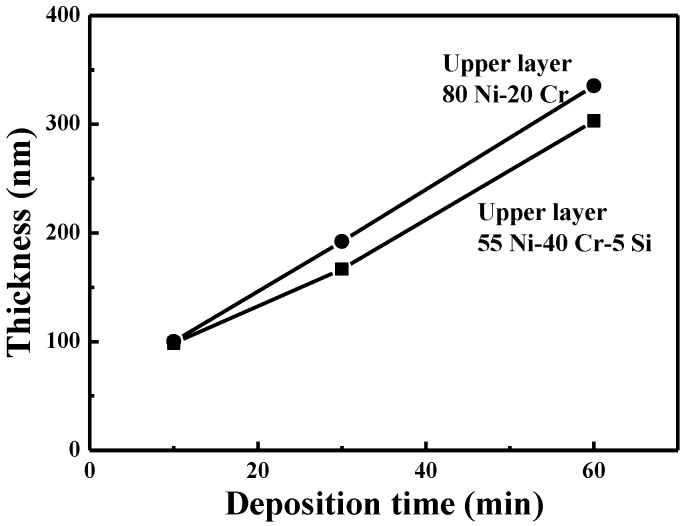
Variations in the thickness of the bi-layer structure as a function of deposition time of the NiCrSi thin films.

The results in Figure 8 show that the thickness of the NiCrSi thin films in the bi-layer structure also increased with the increase of deposition time, but the thin films had different deposition rates, depending on whether they were used as upper or lower layers. We also investigated how the deposition time of the NiCrSi thin films would affect the thicknesses of the two NiCr-NiCrSi bi-layer structures’ thin films, and the results are shown in Figure 9. When the NiCr thin film deposited for 10 min was used as the upper layer and the NiCrSi thin films deposited for 10, 30, or 60 min were used as lower layer, the thickness of the bi-layer thin-film resistors (or the NiCrSi thin films) was about 100.2 nm (33.8 nm), 192.2 nm (125.9 nm), and 335.5 nm (270 nm). When the NiCr thin film deposited for 10 min was used as the lower layer and the NiCrSi thin films deposited for 10, 30, or 60 min were used as upper layer, the thickness of the NiCrSi thin films deposited for 10, 30, or 60 min (or of the NiCrSi thin films) was about 98.5 nm (31.6 nm), 166.6 nm (100.8 nm), and 303 nm (236 nm). We expected that as the deposition time of the NiCrSi thin films increased, so too would the thickness of the bi-layer structures’ thin films. Our results also suggest that when the NiCr thin films were used as the lower layer, the NiCrSi thin films had a lower deposition rate.

**Figure 10:**
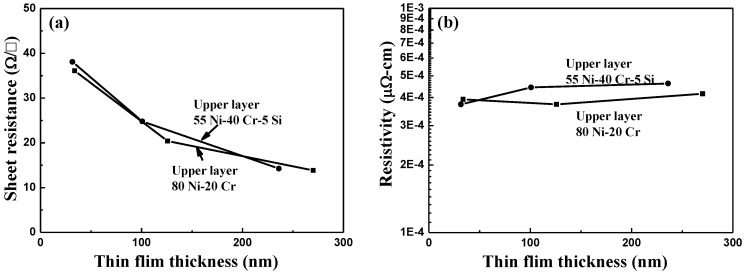
Variations in the (**a**) resistance and (**b**) resistivity of the bi-layer thin-film resistors as a function of the NiCrSi thin films’ thickness.

We also recorded the bi-layer thin-film resistors’ resistance using the four-point probe method and derived the resistivity from the resistance using a measurement of the thin films’ thicknesses, as shown in Figure 9. In addition, we examined the effect of the NiCrSi deposition time on the resistance and resistivity of the bi-layer thin-film resistors as a function of the NiCrSi thin films’ thickness, as Figure 10 shows. The results in Figure 10 indicate two important points: (i) regardless of whether the NiCrSi thin films were used as the upper or the lower layer, the resistance of the bi-layer structure decreased as the NiCrSi thin films’ thickness (or the deposition time) increased; (ii) the resistivity of the bi-layer structure remained stable even if the NiCrSi thin films’ thickness increased. These results suggest that thin-film resistors with stable resistances can easily be achieved by using a bi-layer structure.

**Figure 11:**
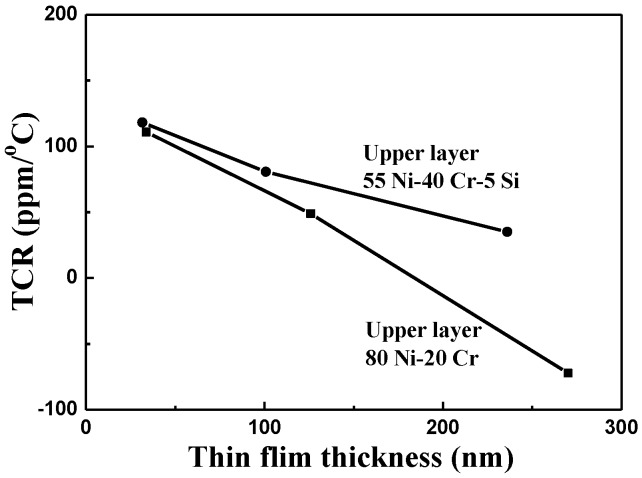
Temperature coefficient of resistance of bi-layer thin-film resistors as a function of our NiCrSi thin films’ thickness.

Figure 11 presents the TCR values of our bi-layer thin-film resistors as a function of the NiCrSi thin films’ thickness. We found that the deposition time of the NiCrSi thin films in the two different structures had a large effect on the TCR values of the NiCr-NiCrSi bi-layer thin-film resistors. When the NiCrSi thin films were used as the upper layer and their thickness increased from 31.6 nm to 236 nm, the TCR value of the bi-layer thin-film resistors dropped from 118.1 to 35.1 ppm/°C, coming close to zero ppm/°C as the NiCrSi thin films’ thickness increased. When the upper layer was the NiCr thin films and the thickness of NiCrSi thin films increased from 33.8 to 270 nm, the TCR value of the bi-layer thin-film resistors shifted from 110.8 to −72.4 ppm/°C. Hence, the TCR changed from close to 0 ppm/°C to a negative value as the NiCrSi thin films’ thickness increased. Compared with the thickness of the bi-layer structure shown in Figure 9, when the NiCrSi thin films were used as the lower (upper) layer, their thickness increased to 33.8 (31.6) nm, 125.9 (100.8) nm, and 270 (236) nm, respectively.

Except the effect of the thickness of the NiCrSi thin films, the material in contact with the Ag electrode is another possible factor affecting the TCR value in these bi-layer thin-film resistors. Many scattering effects are believed to influence the resistivity of bi-layer thin-film resistors, including the surface scattering effect, the grain boundaries (or interface) scattering effect, the uneven or rough surfaces scattering effect, and the impurities scattering effect [16]. In a thin-film material, if, as proposed, the thin films have smooth or even surfaces, then surface scattering is believed to be the main factor affecting their electrical properties. Even the splitting is not really observed in the bi-layer thin films shown Figure 8, the variations of TCR values are apparently influenced by the stacking method and thickness of NiCrSi thin films. If the lower layer is conducted with the Ag electrode, the ohmic conduction mechanism will dominate due to the contact between the lower-layer materials (NiCr or NiCrSi thin films) and the Ag electrode. We believe that an interface layer exists between the upper-layer and lower-layer thin-film materials, so an interface scattering effect and a rough surface scattering effect will happen at the contact boundaries, causing the variations in the TCR values of the bi-layer thin-film resistors. Those results suggest that the thickness of the NiCrSi thin films will dominate the TCR values in such bi-layer thin-film resistors. Those results also suggest that the bi-layer structure is an important technology for developing thin-film resistors with TCR values close to 0 ppm/°C.

## 4. Conclusions

In our bi-layer structure, regardless of whether NiCr thin films were used as the lower layer or the upper layer, their thickness was around 65 nm. As the deposition time was 10 min, the thickness of the NiCrSi thin films in these bi-layer structures was similar to the thickness in a single-layer structure. Their resistances decreased as the deposition time of the NiCrSi thin films increased. The TCR values of our as-deposited NiCr and NiCrSi thin-film resistors were in the range of 197.2 to 230.1 ppm/°C and −106.4 to −153.3 ppm/°C, respectively. For the bi-layer thin-film resistors, as the deposition time of the NiCrSi thin films increased from 10 to 60 min, the thickness increased from 31.6 to 236 nm when we used them as the upper layer, and from 33.8 to 270 nm when we used NiCr thin films as the upper layer. As the deposition time of the NiCrSi thin films increased from 10 to 60 min, the TCR value changed from 118.1 to 35.1 ppm/°C when we used NiCrSi thin films as the upper layer, and from 110.8 to −72.4 ppm/°C when we used NiCr thin films as the upper layer.
